# Positive Urine Morphine Test in a Chinese Patient Receiving Methadone Maintenance Treatment After Eating Hot Pot: A Case Report

**DOI:** 10.3389/fpsyt.2020.00637

**Published:** 2020-07-03

**Authors:** Jun Ma, Yafei He, Kuan Zeng, Xuebing Liu

**Affiliations:** ^1^ Affiliated Wuhan Mental Health Center, Tongji Medical College, Huazhong University of Science & Technology, Wuhan, China; ^2^ Research Center for Psychological and Health Sciences, China University of Geosciences, Wuhan, China

**Keywords:** poppy shells, hot pot, urine morphine test, methadone maintenance treatment, case report

## Abstract

Poppy shells contain opioids. It is a popular, but illegal spice in China. If these shells are added to food, urine morphine test of the patients involved in methadone maintenance treatment (MMT) can turn out positive. A 44-year-old male patient, who had been receiving MMT for 12 years with an extremely good treatment adherence, presented with positive urine morphine test in routine clinical compliance monitoring. However, the patient denied the use of any opioids recently. Coincidently, all of his four family members (none with a history of heroin abuse) showed positive results in urine morphine test. Considering that all these people ate a hot pot together a day before the test added to the speculation that the positive result could be due to the addition of poppy shell to the hot pot. Based on our results, we propose that this unusual phenomenon is worthy of clinical attention when managing patients at MMT clinics.

## Introduction

Opioid abuse is a global public health concern (1). In China, although the most-frequently used drug in the recent time has been synthetic drugs, opioids, mainly heroin, remain widely prevalent in use, accounting for nearly 40% of all illicit drugs being consumed in China (2). Methadone maintenance treatment (MMT) is an effective treatment for heroin addiction and has been a nationwide strategy for treating Chinese individuals with heroin dependence in communities (3). MMT is mainly applicable to heroin-dependent patients. Under this program, a certain amount of methadone is administered to the addicted patients daily in a certain community so as to reduce the harm caused by heroin as well as to help them quit their habit and return to the society (4). According to the report from the Chinese Center for Disease Control and Prevention (CDC) in 2016, there were a total of 773 MMT clinics in China, where nearly 159,700 people were undertaking the MMT. Patients with >70% of heroin dependence were accepted by the MMT program. Since the treatment, the health conditions and employment rates of these patients have improved, and the incidences of acquired immune deficiency syndrome (AIDS) and hepatitis C have decreased significantly (5).

However, this management model has some limitations, such as the continued use of heroin by the patient. To avoid this non-adherence behavior, Chinese patients under MMT are required to receive urine morphine test once a month. A positive urine morphine test generally indicates the use of heroin by the patient in a recent few days, suggesting a failure to adhere to the MMT. Furthermore, because of the interaction between heroin and methadone, the adjunctive use of heroin may lead to fatal methadone overdose.

## Case Report

This report presents the case of a Chinese MMT patient who was found to be positive in the urine morphine test although he did not have a history of heroin use since a few days before the test.

The medication history and medical events were summarized in **Table 1**: Timeline. A 44-year-old man, Mr. Zhang, was undertaking the MMT at the Wuhan Mental Health Center. In the early 2001, depressed by failure in his business, the patient started smoking heroin with the encouragement of his friends. At first, he adopted a “curious and try” attitude. The heroin dosage was relatively small at the start, approximately 0.1 g/time, 3–4 times a day. However, by the end of 2001, the daily dose of heroin inhaled by the patient reached about 1.2 g, since then, the situation of drug use continued to worsen. In the first half of 2002, he started taking heroin intravenously, with a daily dose of approximately 0.6–1.2 g, divided into 3–4 injections. Compulsory detoxification was requested by the government from May 2003 to August 2004. From June 2007, the patients began to receive 60 ml/d of MMT, and showed good treatment compliance. In 2009, the patient was discharged after a good recovery and he continued to adhere to the MMT treatment protocol in the outpatient clinic, and gradually resumed his business.

**Table 1:** Presentation of medication history and medical events.

Timeline	
In the early 2001	inhale heroin 0.1 g/time, 3–4 times a day
The end of 2001	the daily dose of heroin inhaled about 1.2 g
In the first half of 2002	the daily dose of heroin intravenously 0.6–1.2 g, 3–4 times a day
May 2003 to August 2004	compulsory detoxification
June 2007	receive 60 ml/d of MMT
October 22, 2019	positive urine test
October 23, 2019	negative urine test

The compliance of the patient to MMT was extremely good. However, on October 22, 2019, he was tested and found positive for the urine morphine test. Mr. Zhang was very surprised about the result, and the patient denied using any opioids. On that day, the patient's Clinical Opiate Withdrawal Scale (COWS) score was 2 and the visual analogue scale (VAS) score was 0. Moreover, our team reviewed the patient's outpatient medical records in the past year. As per the record, in the past year, the common health indicators of the patient in the outpatient department were generally normal, such as electrocardiogram (ECG), blood routine, liver function, renal function, and blood glucose; the patient visited our clinic nearly every day, and the result of his urine morphine test was always negative.

After detailed inquiry, this patient recalled that he and his family had eaten hot pot at a restaurant a day before the positive testing. People who ate with him included his parents, wife, and son. After providing informed consent, we obtained the blood samples of the patients' four family members and conducted a urine morphine test on the samples on the same day. All the results obtained were positive. On October 23, 2019, the urine test was performed again for the entire family, and all the results came negative. Later, after repeated inquiries by the patient, the hot pot owner privately admitted that poppy shells were added to the hot pot soup served to them. Therefore, we speculated that the positive test results of the entire case family were a result of consumption of poppy shells-added hot pot. Following a 3-month outpatient follow-up, the patient's urine test results came negative. Throughout the follow-up, we continued to encourage and psychologically support the patient, making him believe that he is a model of successful detoxification who may hopefully exchange his experience with other needy people. Fortunately, all the patients and their families were very happy to know of the successful treatment outcome.

## Discussion and Conclusions

Hot pot is one of the most popular Chinese foods. As a Chinese traditional cooking skill, poppy shell or its powder is occasionally added to hot pot, based on an old rumor that poppy shells make food more delicious. Furthermore, in this way, even in restraints, the dining experience of diners is improved. As a result, hotpot restaurants attract increased customer traffic. Generally speaking, diners believed that eating food containing poppy shells produce a certain level of euphoria experience as a result of the addition of a small amount of opioid ingredients in the meal duo to the reward effect of opioids. Poppy shell has a long history of medicinal use in China, and it continues to be used widely in clinical practices. It is the only Chinese medicine decoction substance in the national narcotic drug management catalog (6), although its long-term use is known to induce addiction (7). The toxic reactions caused by poppy shells are mainly attributable to opioid alkaloids, which include more than 30 types of alkaloids such as morphine, codeine, narcotine, papaverine, and thebaine. Morphine and codeine account for approximately 20% of the total alkaloids, of which morphine accounts for 7–15%, followed by codeine, which accounts for approximately 0.3–4% (8, 9). Past studies have reported that morphine and codeine are the toxic components of poppy husks. The toxic effects and the pharmacological activities of poppy husks are related to those of alkaloids contained in them; both morphine and codeine are the main active and toxic components (10, 11).

The repeated and complicated detoxification process for opioid addicts and the characteristics of addicted patients who often lie makes it important to remain vigilant in clinical practices to identify addicts who are aware that consumption of foods containing poppy shells can give positive urinary inflammatory test and use this fact as a cover to secretly consume heroin. According to the relevant provisions of Article 33 and Article 47 of Chapter IV of the Drug Law of the People' Republic of China, the duration for community drug rehabilitation for drug addicts is 3 years or even 2 years for compulsory isolation. For the staff involved in clinical practice in the process of drug law enforcement, it is critical to establish a process to distinguish whether positive urinalysis is caused by heroin to avoid awarding erroneous punishment. The metabolites of heroin are morphine and 6-O-monoacetylmorphine (12). Past studies have revealed that, for a single use of heroin, the longest detection time of 6-O-monoacetylmorphine in the blood is 6 h and the shortest is only 1.5 h (for a dose of 1.3 mg). Its further metabolite, morphine, cannot be detected in the blood for 24–36 h; therefore, a single use of heroin or poppy shell is usually not detected after 24–36 h (13). For patients who repeatedly use heroin, the time for a test to turn negative is longer, usually about 72 h (14). The above facts are an important basis to distinguish between testing for heroin and poppy shell foods. In our case report, because of consuming hot pot supplemented with poppy shell, the patient and his family all showed positive results in the urine morphine test. However, because the amount of opium intake was extremely small, their urine morphine test results became negative immediately after 1 d. This clinical phenomenon is different from that of those who used heroin, whose test results turn negative 3–5 d after the injection due to the large amount consumed. On this basis, we infer that the possibility of heroin-patients relapsing is extremely small. After further confirmation by the owner of the hot pot restaurant, we ruled out the above inference. We believe that the right treatment approach can promote the patient's will to continue adherence to MMT treatment and to enhance his confidence to actively overcome his drug addiction.

The Food Safety Law of the People's Republic of China has specified that the addition of poppy shell to the food in any form is illegal. In fact, in 2008, the Ministry of Health of China listed poppy shells under the “List of Non-edible Substances and Food Additives that May Be Illegally Added in Foods (First Batch)”. Adherence to this statement prohibits illegal additions of poppy shells to foods and strictly regulates their use. In China, the catering industry is quite prosperous. As hot pot is much loved by the Chinese people, it is served quite frequently on the streets of cities. It is impossible to perform routine urinalysis on the general population to determine whether poppy shells exists in the hot pot served across cities unless the patients receiving MMT treatment are followed up at the drug treatment clinic. Although prohibited by law, some people still prefer to take the risk of using poppy shells for economic benefits. Therefore, we suggest that the government should strengthen the propaganda of the legal system and improve self-discipline among catering practitioners, as well as actively develop rapid detection technology for hot pot soup to identify the illegal addition of poppy shells in hot pot, strengthen the punishment for illegal abuse of poppy shell, and minimize the harm of opioids to the population through various treatment measures.

For MMT patients, who are considered extremely special in China, if the urine test is positive, they should be subjected to stricter scrutiny so as to confirm possible relapse or failure of the community detoxification process. This approach leads to psychological harm and confidence frustration, especially in patients with good MMT community treatment compliance and relatively complete social function. Therefore, great attention should be paid to such patients' diet and medication and weekly urine tests should be conducted for them according to the clinic requirements. In case of accidental positive results, we must look for the cause of positive results and clarify through various processes in order to avoid unnecessary hassles for the patients.

## Data Availability Statement

The original contributions presented in the study are included in the article/supplementary material; further inquiries can be directed to the corresponding author.

## Ethics Statement

Written informed consent was obtained from the individual(s) for the publication of any potentially identifiable images or data included in this article.

## Author Contributions

XL made substantial contributions to conception and design of the study. JM and KZ drafted and revised the manuscript. YH was responsible for collecting case information and subsequent follow-up interviews. XL gave final approval of the version to be published.

## Funding

This study was funded by the Wuhan Health and Family Planning Commission (WX17A07, XL, PI) and the Wuhan Health Commission (WX19Y12, JM, PI).

## Conflict of Interest

The authors declare that the research was conducted in the absence of any commercial or financial relationships that could be construed as a potential conflict of interest.
